# Life history change in response to fishing and an introduced predator in the East African cyprinid *Rastrineobola argentea*

**DOI:** 10.1111/j.1752-4571.2012.00245.x

**Published:** 2012-11

**Authors:** Diana M T Sharpe, Silvester B Wandera, Lauren J Chapman

**Affiliations:** 1 Department of Biology, McGill UniversityMontréal, Québec, Canada; 2 National Fisheries Resources Research Institute (NaFIRRI)Jinja, Uganda

**Keywords:** anthropogenic stressors, contemporary phenotypic change, dagaa, fisheries-induced evolution, invasive species, life history evolution, mukene

## Abstract

Fishing and introduced species are among the most important stressors affecting freshwaters and can also be strong selective agents. We examined the combined effects of commercial fishing and an introduced predator (Nile perch, *Lates niloticus*) on life history traits in an African cyprinid fish (*Rastrineobola argentea*) native to the Lake Victoria basin in East Africa. To understand whether these two stressors have driven shifts in life history traits of *R. argentea*, we tested for associations between life history phenotypes and the presence/absence of stressors both spatially (across 10 Ugandan lakes) and temporally (over four decades in Lake Victoria). Overall, introduced Nile perch and fishing tended to be associated with a suite of life history responses in *R. argentea*, including: decreased body size, maturation at smaller sizes, and increased reproductive effort (larger eggs; and higher relative fecundity, clutch volume, and ovary weight). This is one of the first well-documented examples of fisheries-induced phenotypic change in a tropical, freshwater stock; the magnitude of which raises some concerns for the long-term sustainability of this fishery, now the most important (by mass) in Lake Victoria.

## Introduction

A growing body of evidence suggests that anthropogenic stressors may be precipitating rapid phenotypic change as species struggle to adapt to human-dominated environments (Reznick and Ghalambor 2001; Stockwell et al. 2003; Hendry et al. 2008). However, much uncertainty remains regarding the rate, limits, and ubiquity of such responses, as well as the mechanisms that underlie them. This uncertainty compromises our ability to predict species’ persistence in the face of continuing anthropogenic disturbance and to inform management decisions. In freshwater systems throughout the world, fishing and introduced species are among the most important stressors, with significant ecological and socio-economic impacts (Sala et al. 2000; Beeton 2002). In addition to their immediate ecological effects, they can also be important selective agents, with the potential to drive contemporary phenotypic change in native prey/harvested species (Palumbi 2001; Hendry et al. 2008; Darimont et al. 2009). The potential for fishing to drive evolutionary changes in the life histories of exploited fish populations has long been recognized, given that fisheries: (i) are often highly selective, (ii) frequently impose mortality much greater than natural mortality, and (iii) that many life history traits potentially under fisheries selection are heritable (Stokes and Law 2000; Heino and Godo 2002). In the past decade, several lines of evidence have helped guide an emerging consensus that fisheries-induced evolution is not only theoretically possible, but likely common in nature. These include: experimental evidence that artificial selection mimicking harvest can drive rapid evolution in life history traits (Conover and Munch 2002; Walsh et al. 2006); direct measurements of fisheries selection on wild populations (e.g., Carlson et al. 2007; Kendall et al. 2009; Olsen and Moland 2010); and long-term field data from numerous commercial fish stocks showing phenotypic trends consistent with fisheries-induced evolution (reviewed in Trippel 1995; Law 2000; Hutchings and Baum 2005; Jørgensen et al. 2007; Sharpe and Hendry 2009). However, studies to date have been heavily biased toward a small number of temperate marine fishes (Sharpe and Hendry 2009), with none looking at fisheries-induced change in tropical, freshwater species. This is a critical research gap for several reasons. First, tropical fishes often have different life histories (e.g., several species breed throughout the year) and so might respond differently to harvest selection. Second, many inland tropical fisheries are artisanal, with different types of gear employed and little formal management, likely resulting in different and heterogeneous patterns and intensities of fishery selection. Third, inland tropical fisheries play an extremely important role in ensuring local food security, particularly in developing areas (Dugan et al. 2010), making it important to assess the prevalence of fisheries-induced evolution in these areas and determine how it can guide management practices.

Like fishing, biologic invasions, particularly of predators, can be a potent form of novel selection. There is growing interest in understanding the extent to which selection pressures induced directly or indirectly by invasive species may be driving plastic and/or evolutionary changes in native prey (Mooney and Cleland 2001; Strauss et al. 2006; Carroll 2007). Native prey may adapt to introduced predators via the adoption of novel predator-avoidance behaviors (e.g., Kiesecker and Blaustein 1997; Losos et al. 2004), the evolution of morphological defenses (e.g., Vermeij 1982), and/or altered life history tactics (e.g., Fisk et al. 2007). Beyond these few examples, however, we still know relatively little about the evolutionary consequences of biotic invasions (Strauss et al. 2006), especially in freshwaters, which are among the most heavily invaded ecosystems (Strayer 2010).

In this study, we examined the combined effects of fishing and an introduced predator on life history traits in the cyprinid fish, *Rastrineobola argentea*, native to the Lake Victoria basin in East Africa. Lake Victoria is the largest tropical lake in the world and Africa’s most important inland fishery. It has undergone multiple anthropogenic changes over the past century, including overfishing of native fish stocks, eutrophication, and the introduction of several non-native fishes in the 1950s and 60s, most notably the predatory Nile perch, *Lates niloticus* (see reviews by Kaufman 1992; Balirwa et al. 2003; Chapman et al. 2008). Together these stressors have led to the dramatic decline of many native fishes and the extinction of hundreds of Lake Victoria’s endemic haplochromine cichlids (Ogutu-Ohwayo 1990b; Witte et al. 1992; Seehausen et al. 1997; Goudswaard et al. 2008). However, one native fish species has not only persisted, but thrived through this period of ecological upheaval: the small pelagic cyprinid *Rastineobola argentea*, known locally as mukene in Uganda, omena in Kenya, and dagaa in Tanzania.

*Rastrineobola argentea* has experienced increased mortality over the past half century from two major anthropogenic sources: predation from the introduced Nile perch and commercial fishing. Predation pressure from the Nile perch likely peaked in the mid 1980s when, after depleting its preferred prey, the haplochromine cichlids, the Nile perch began to prey on *R. argentea* throughout its invaded range (Hughes 1986; Ogutu-Ohwayo 1990a, 1993; Mbabazi 2004). Nile perch still prey on *R. argentea* today, although to a lesser extent (Budeba and Cowx 2007; Paterson and Chapman 2009). This likely reflects both an overall decline in the biomass of Nile perch, likely due to overfishing (Ogutu-Ohwayo 2004; Mkumbo et al. 2007; Muhoozi 2008), and a shift in the Nile perch diet back to the haplochromines cichlids, which appear to be undergoing a limited resurgence in some parts of Lake Victoria (Witte et al. 2000). Commercial fishing for *R. argentea* began in the late 1980s in Uganda, and landings have increased almost exponentially since then, making it now the most important fishery (by mass) in Lake Victoria (NaFIRRI, 2008). To test whether these two novel stressors have driven any shifts in life history traits of *R. argentea*, we used a combined approach, testing for associations between life history phenotypes and the presence/absence of stressors both spatially and temporally. For the spatial approach, we asked how life history traits of *R. argentea* varied across 10 lakes in Uganda that differ in the presence/absence of introduced Nile perch and *R. argentea* fisheries. For the temporal approach, we asked how life history traits of *R. argentea* have changed over the past four decades in Lake Victoria, following the introduction of the Nile perch and the onset of commercial fishing.

Classical life history theory predicts that increased mortality on adults will select for reduced age and size at maturation and increased reproductive effort, whereas increased mortality on juveniles should have the opposite effect. If mortality is uniformly distributed across age classes, no evolution is expected to occur (Gadgil and Bossert 1970; Law 1979; Michod 1979). However, more recent models of life history evolution argue that increased mortality, even if applied to both mature and immature individuals, will select for earlier maturation at smaller sizes (Law and Grey 1989; Abrams and Rowe 1996; Ernande et al. 2004).

We suggest that this latter prediction is most likely to apply to *R. argentea*, as the commercial fishery targets a broad range of sizes that includes both mature and immature individuals (Wandera 1992; Taabu 2004; D. M. T. Sharpe and L. J. Chapman unpublished data). There have been no direct measurements of the size-selectivity of Nile perch predation on *R. argentea*, but several lines of evidence suggest that both mature and immature *R. argentea* are regularly consumed by Nile perch after their ontogenetic dietary shift to piscivory (Ogutu-Ohwayo 1985, 2004; NaFIRRI 2004; Katunzi et al. 2006; but see Wanink 1998).

## Materials and methods

### Study sites

**Figure 1:**
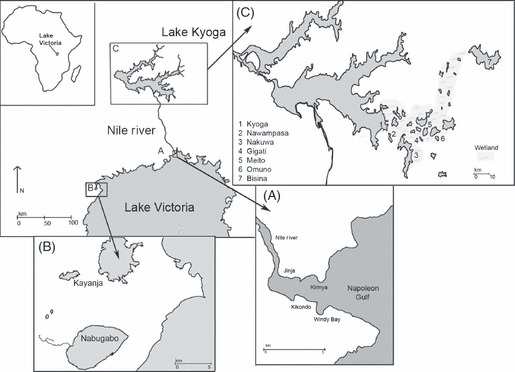
Map of study sites within the Lake Victoria basin of East Africa, with sampling regions enlarged. The 10 lakes sampled for this study were: Victoria (A), Nabugabo (B), Kayanja (B), Kyoga (C), Nawampasa (C), Nakuwa (C), Gigati (C), Meito (C), Omuno (C) and Bisina (C). All contemporary (2008–2010) and historical (1966–2000) sampling on Lake Victoria was carried out in the Napoleon Gulf (A), a large sheltered bay on the northern shore of Lake Victoria, just upstream of the source of the Victoria Nile. The catchment is densely populated (587 people/km^2^) and encompasses Jinja, the second largest city in Uganda and a major national industrial centre. Lakes Nabugabo and Kayanja (B) are small satellite lakes on the Northwestern shore of Lake Victoria. The Lake Kyoga system (C) is comprised of over a dozen small, shallow lakes interconnected by extensive wetlands, which together span over 4700 km^2^ across the Ugandan districts of Kamuli, Pallisa, Kumi, and Soroti. The wetlands of the Kyoga system are dominated by dense stands of papyrus, *Cyperus papyrus*, and are considered to be one of the remaining pristine wetland areas in Uganda, due to their remote location and relatively low population densities. The Kyoga system is characterized by strong seasonal flooding during the two rainy seasons, and so water levels and connectivity among lakes may shift over time.

Our study focussed on 10 different lakes located in three parts of the Lake Victoria basin in Uganda, East Africa (Fig. 1). These included Lake Victoria, two satellite lakes on the northwestern shore of Lake Victoria, and seven lakes from the Kyoga lakes system, located approximately 150 km north of Lake Victoria. Our sampling covered all of *R. argentea*’s known distribution in Uganda, as well as uncovering six previously unknown populations. Lakes were classified into three perturbation levels based on Nile perch invasion history and the level of fishing pressure for *R. argentea* (Table A1 in Appendix S1). The perturbation categories were lakes without introduced Nile perch or fishing for *R. argentea* (‘Unperturbed’, *n* = 3), lakes with introduced Nile perch but little or no fishing for *R. argentea* (‘Nile Perch’, *n* = 5), and lakes with both introduced Nile perch and commercial *R. argentea* fisheries (‘Nile Perch and Fishing’, *n* = 2). Some native fishes are also known to consume *R. argentea*, including the catfishes *Clarias gariepinus*, *Schilbe intermedius* and *Synodontis victoriae* and the mormyrid *Gnathonemus victoriae* (Mbabazi 2004). Although data on the distribution and diet of these species in our study lakes are sparse, the available evidence does not suggest that they constitute a major source of mortality for *R. argentea* in most of our study lakes (Table A1 in Appendix S1). Several native birds are also known to feed on *R. argentea* in the Tanzanian portion of Lake Victoria, including the pied kingfisher *Ceryle rudis*, the great cormorant *Phalacrocorax carbo lucidus*, and the long-tailed cormorant *Phalacrocorax africanus* (Wanink and Goudswaard 1994; Wanink 1996). We do not know to what extent these avian predators may target *R. argentea* in Ugandan lakes.

### Field collections

*Rastrineobola argentea* were collected from the 10 lakes described above during three field expeditions carried out during the dry season months of May-July in 2008, 2009, and 2010. A variety of fishing gears were used to capture as broad of a size range of fish as possible, although not all gears were successful in all lakes. In each lake, 2–4 sets of experimental gill-nets (nylon monofilament, ½″ (12.7 mm) stretched mesh, 1.83 m deep x 12.2 m long) were set overnight (approximately 16 h) in the pelagic zone of the lake. We also attempted surface seining during the day time with a 5-mm mesh lampara net in the pelagic zone. On a few lakes (Nabugabo, Nawampasa, and Meito), we fished for *R. argentea* at night using the local fishing methods, which involves concentrating schools of *R. argentea* at the surface using light attraction from kerosene pressure lanterns, and then trapping them with a 5-mm mesh lampara net. On Lake Victoria, we collected *R. argentea* directly from fishers who were fishing at night on the Napoleon Gulf, about 1 km offshore from Kikondo (Fig. 1). After capture, fish were euthanized in either buffered MS-222 (2008 and 2009) or clove oil (2010) and immediately preserved in 10% formalin.

At each lake, we also collected environmental data at three replicate sites in the morning and in the afternoon. These data included water depth, water transparency (Secchi depth, m), and water temperature (°C) and dissolved oxygen (mg/L), measured with a Polaris dissolved oxygen meter.

### Measurement of life history traits

Preserved *R. argentea* were measured (standard length to the nearest mm), weighed (to the nearest 0.01 g), and then dissected to determine sex and maturity status. Gonad maturity was macroscopically assessed based on a seven-point scale previously developed for *R. argentea* (S. B. Wandera, unpublished data). The ovaries of mature (Stage V and VI) females were removed and weighed (wet weight, to the nearest mg). Somatic weight was calculated by subtracting the ovary weight from the total body weight. Total fecundity was estimated using the gravimetric method (Hunter et al. 1985). We counted the number of yolked oocytes in three weighed subsamples taken from the center, posterior, and anterior regions of the left ovary of mature (Stage V and VI) females. These counts were then extrapolated to the entire ovary by multiplying the average oocyte density by the total weight of the ovary.

Egg volume was estimated by measuring two perpendicular diameters on 20 randomly selected eggs from mature (Stage V and VI) females, using the image measurement software Motic (v. 2.0, 2003). We used the average of these two diameters (*d*) to estimate the volume of the egg, approximated as a sphere: 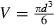. These 20 measurements were then averaged to obtain one estimate of mean egg volume per female. Clutch volume was calculated as the product of mean egg volume and total fecundity. Finally, we also recorded the presence of intestinal parasites, as these are known to grow to such a large size as to partially or completely impair gonad development in *R. argentea* (Cowx et al. 2008). Nematode parasites were found in a moderate proportion of fish from Lake Kayanja (19%), and cestode parasites were found in a small proportion of *R. argentea* from Lakes Victoria (4.6%) and Kyoga (0.2%). *R. argentea* from all other lakes were found to be free of intestinal parasites. Parasitized females were excluded from all analyses on reproductive traits. We did not undertake an aging study of *R. argentea*, although there is evidence for daily ring deposition in this species (Njiru et al. 2001), and this could be an important avenue for future work.

### Statistical analysis

#### Spatial analysis

##### Body size

To examine variation in mean body size across lakes and perturbation levels, we compared the standard length of a random sample of fish caught in the least size-selective of our fishing gears (5-mm lampara net). This was done using a nested anova, with lake as a fixed factor nested within perturbation category. Lake was considered as a fixed factor in all of our analyses because, to the best of our knowledge, our sampling covered the entirety of *R. argentea’*s range in Uganda. That is, lakes were not chosen randomly as examples of particular perturbation regimes; rather, we sampled all lakes known to contain *R. argentea* and then grouped them based on the stressors that they had been exposed to.

Two lakes (Kayanja and Nakuwa) were excluded from the body size analysis because we did not manage to catch *R. argentea* using the lampara net in these lakes. Five lakes were sampled with lampara nets in more than 1 year (Gigati, Bisina, Kyoga, Nawampasa, and Victoria); the rest were only sampled in 2010. For four of the five lakes that were sampled in multiple years, there were small, but statistically significant differences between years (results not shown). For this reason, we examined overall differences in body size across lakes in two ways: with sampling years considered separately and sampling years pooled.

##### Length at 50% maturity

To compare length at 50% maturity (*L*_50_) across lakes and perturbation levels, we used fish collected in 2010, as this was the year with the most robust sample size, and the greatest number of lakes sampled. Two lakes were excluded from the analysis, one because of low sample size (Nakuwa), and the other because we could not detect a significant association between body size and the probability of maturation (Kayanja). Length at maturity was estimated for females only, as macroscopic staging of male gonads in such small fish is not very reliable.

To test for differences in *L*_50_, we ran a nested generalized linear model (GLM) with maturity status (i.e., mature or immature) as a binomial response variable and the following as explanatory variables: perturbation level, lake nested within perturbation level, and standard length. We assumed a binomial error distribution and used a logit link function. We tested the significance of each effect by sequentially removing terms from the model, and testing whether such deletions caused a significant decrease in the log-likelihood of the model, using the likelihood ratio test. Lake-specific values of *L*_50_ were calculated by setting the probability of maturation to 0.5 in the regression equation for each lake, which simplifies to:


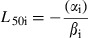


where α_i_ and β_i_ are the intercept and slope, respectively, for lake i. To test for pairwise differences among specific perturbation levels, we performed an anova on the lake-level estimates of *L*_50_, followed by *post hoc* Tukey’s tests. As the assumptions of normality and homoscedasticity were not well met with so few data points, we also repeated the analysis using the nonparametric Kruskal–Wallis test.

##### Reproductive traits

We compared reproductive traits (fecundity, egg volume, clutch volume, and ovary weight) across lakes using nested ancova, with lake as a fixed factor nested within perturbation level and somatic body weight as a covariate. Body weight data were missing for five fish in the dataset, and so were estimated for these individuals using lake-specific length–weight relationships derived from data for all other fish from that lake (*R*^2^ values for these length–weight relationships were always >0.98). When necessary, variables were log-transformed to improve normality and homogeneity of variances. For lakes that were sampled in multiple years, we pooled sampling years because visual inspection of the data showed that there were no major differences among years and pooling improved sample size.

##### Across-lake trait covariation

We tested for across-lake trait covariation using Pearson correlation tests on size-standardized trait means. In particular, we were interested in testing for: (i) covariation between mean length/*L*_50_ and size-adjusted reproductive effort (clutch volume and ovary weight adjusted to a common body mass), and (ii) trade-offs between egg size and egg number.

##### Assessing the effect of environmental variables

We ran a separate series of tests to investigate how results from the models described above would be affected by inclusion of lake-specific environmental data (lake area, lake depth, temperature, dissolved oxygen, and water transparency). Several of these environmental variables were strongly intercorrelated, so we first performed a principal components analysis on scaled, centered lake means to reduce dimensionality of the data. The first three principal components (PCs) explained 87% of the variance, so these were retained to be used as explanatory variables in our models. For each trait, we built a GLM that included perturbation level, environmental PCs 1–3, and somatic body weight as covariate, where appropriate. Our interest here lay in testing whether any effects of perturbation level observed in the nested models above remained if we replaced lake with specific environmental data.

#### Temporal analysis

##### Historical data

We were able to obtain historical data and/or specimens of *R. argentea* from Lake Victoria for six different time periods spanning the past four decades (Table B1 in Appendix S2). Data from 2000 and 2003 were pooled to increase sample size. All historical samples were collected from the Napoleon Gulf of Lake Victoria, which is where we carried out our contemporary (2008–2010) sampling as well. For Lake Kyoga, we were able to obtain historical data from 1991 (Table B1 in Appendix S2). These data were based on specimens collected at Bukungu Landing, about 50 km away from the Lyingo Landing of Lake Kyoga, where we carried out our contemporary (2008–2010) sampling. Using this combination of museum specimens and unpublished historical data, we were able to examine temporal changes in all life history traits, except for standard length, because different fishing gears were used to capture *R. argentea* in different years, so size distributions were not directly comparable.

##### Length at 50% maturity

To test for variation in length at maturity (*L*_50_) across time periods in each lake, we used the same approach as for the spatial analysis outlined above. We first ran a GLM with maturity status (i.e., mature or immature) as a binomial response variable and the following as explanatory variables: standard length, year, and the interaction between SL and year. We then used the parameters from this model to estimate *L*_50_ for each year, as detailed above.

##### Reproductive traits

In Lake Victoria, we compared fecundity of *R. argentea* from three key time periods: (i) immediately following the Nile perch introduction into Lake Victoria but before its population boom (1966), (ii) post-Nile perch boom, but before the expansion of the *R. argentea* fishery (1992), and (iii) during the decline of the Nile perch population and large-scale development of the *R. argentea* fishery (2008–2010). In Lake Kyoga, we were only able to examine fecundity for two time periods, both post-Nile perch introduction, but one occurring before (1991) and one after the development of Lake Kyoga’s *R. argentea* fishery (2008–2010). Variation in fecundity through time was explored using separate ancovas for each lake, with log-transformed fecundity as the response variable, and year as a categorical explanatory factor. Only standard length was recorded across all time periods, so we used this as the body size covariate in our analyses. Analyses were restricted to mature (Stage V and VI), nonparasitized females.

Data on egg traits of *R. argentea* were only available from Lake Victoria, and only for two time periods: 1966 and 2008–2010. We compared egg traits (mean egg volume, clutch volume, and ovary weight) across years using ancova, with year as a categorical explanatory factor, and somatic body weight as a covariate. We tested for interactions between year and somatic weight, but removed them when they were not significant to estimate adjusted means. When necessary, variables were log-transformed to improve normality and homogeneity of variances. Because our historical data for egg traits were so limited, we included Stage IV, V, and VI females for this analysis to improve our sample size. Trends were very similar (although not always statistically significant) if we did restrict our analysis to Stage V and VI females, as in other analyses.

##### Rates of phenotypic change

Rates of phenotypic change for each trait over time were calculated in darwins as:


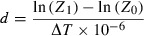


where *Z*_0_ and *Z*_1_ are the trait values at the beginning and end of the time series, respectively, and Δ*T* is the amount of time elapsed, in years. While this metric was initially intended for measuring morphological changes in the fossil record, it has increasingly been applied to the study of contemporary phenotypic change (e.g., Hendry and Kinnison 1999), especially in anthropogenic contexts (Jørgensen et al. 2007; Darimont et al. 2009; Sharpe and Hendry 2009). Another widely used rate metric is the haldane, which is scaled by the generation time of the species and thus more widely comparable across taxa (Hendry and Kinnison 1999). We did not calculate haldanes in our study, because there are no estimates of the generation time for the Ugandan populations of *R. argentea*, and there is also evidence (from studies carried out in the Tanzanian waters of Lake Victoria) that generation time may have changed dramatically over the time frame in question (Wanink 1998).

## Results

### Spatial analysis

#### Body size

**Figure 2:**
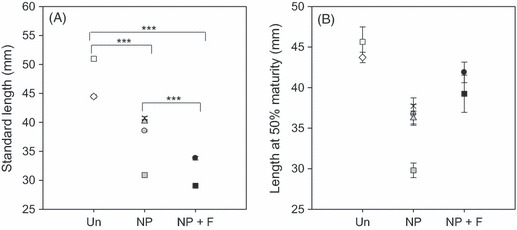
Panel A shows the mean standard length (±1 SE) of *Rastrineobola argentea* caught in 5-mm lampara nets in 2010. Panel B shows the length at 50% maturity (±2 SE) of female *R. argentea* in 2010. The colours represent unperturbed lakes (‘Un’,white), lakes with Nile perch only (‘NP’, grey), and lakes with Nile perch and commercial fishing (‘NP + F’, black). The lakes are: Omuno (white square), Gigati (white diamond), Nawampasa (grey circle), Bisina (grey square), Meito (grey triangle), Nabugabo (grey cross), Kyoga (black circle), and Victoria (black square). For standard length, *post-hoc* Tukey’s tests showed that all group means were significantly different from one another (*P* < 0.001), indicated by ***. For length at maturity, *post-hoc* pair-wise comparisons were not conducted, since each estimate of *L*^50^ is a population-level estimate derived from parameters from the logistic regression. However, we have shown each mean ± 2 SE, which approximate 95% confidence intervals. Thus, pairs of lakes with nonoverlapping error bars can be interpreted as being significantly different at the 0.05 significance level.

The mean standard length of *R. argentea* differed among lakes (*F*_5,2475_ = 369.25, *P* < 0.001) and perturbation levels (*F*_2,2475_ = 1614.12, *P* < 0.001), with *R. argentea* from unperturbed lakes being largest, those from lakes with Nile perch being intermediate, and those from lakes with Nile perch and fishing being smallest (Fig. 2A). These patterns of among-lake variation were very consistent across sampling years, and results were virtually identical whether we looked at data from 2010 only or pooled data from all available sampling years for each lake (results not shown).

#### Length at 50% maturity

The probability of maturation of female *R. argentea* varied significantly across lakes and perturbation levels (Table 1). *L*_50_ was highest in the least perturbed lakes (Gigati, Omuno), intermediate in lakes with Nile perch and fishing (Kyoga and Victoria), and lowest in lakes with Nile perch only (Fig. 2B). An anova of the lake-level estimates of *L*_50_ confirmed the overall significance of perturbation level (*F*_2,5_ = 9.03, *P* = 0.022), with *post hoc* Tukey’s tests indicating that the only significant difference was between the unperturbed and Nile perch categories. Although our sample size was substantially reduced in this latter analysis (because lake-level estimates were used), the model still had high explanatory power (*R*^2^ = 0.70). When we repeated the analysis using the nonparametric Kruskal–Wallis test, perturbation remained significant (Kruskal–Wallis χ^2^_0.05,2_ = 6, *P* = 0.050).

**Table 1:** Analysis of deviance from generalized linear models examining variation in maturation of female *Rastrineobola argentea* across lakes (spatial analysis) and through time in Lake Victoria (temporal analysis). SL refers to standard length (mm)

Analysis	Effect removed	df	Deviance	Residual df	Residual deviance	*P*
Spatial	Perturbation	2	208.32	1001	1165.58	<0.0001
	Lake (Perturbation)	5	149.35	995	585.55	<0.0001
	SL	1	430.68	1000	734.90	<0.0001
Temporal	SL	1	1225.80	2887	2101.7	<0.0001
	Year	8	175.67	2879	1926.0	<0.0001
	Year × SL	8	129.45	2871	1796.5	<0.0001

#### Reproductive traits

**Figure 3:**
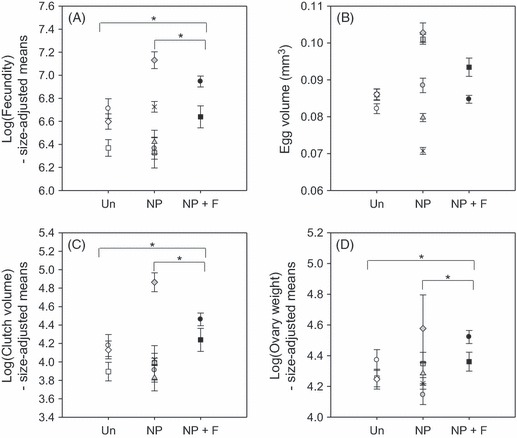
Size-adjusted means (±1 SE) for (A) fecundity, (B) egg volume, (C) clutch volume, and (D) ovary weight of mature (Stage V and VI) female *Rastrineobola argentea.* These are adjusted means from an ancova with log(somatic body weight) as a covariate. The colours represent unperturbed lakes (‘Un’, white), lakes with Nile perch only (‘NP’, grey), and lakes with Nile perch and commercial fishing (‘NP + F’, black). The lakes are: Kayanja (white circle), Omuno (white square), Gigati (white diamond), Nawampasa (grey circle), Bisina (grey square), Nakuwa (grey diamond), Meito (grey triangle), Nabugabo (grey cross), Kyoga (black circle), and Victoria (black square). The * indicate significant (*P* < 0.05) differences between pairs of perturbation categories based on *post-hoc* Tukey’s tests.

Somatic body weight was a significant covariate for fecundity, clutch volume, and ovary weight, but not for egg volume (Table 2). The former traits also varied significantly both across lakes and across perturbation levels (Table 2), being generally lowest in *R. argentea* from unperturbed lakes, intermediate in *R. argentea* from lakes with Nile perch, and greatest in *R. argentea* from lakes with Nile perch and fishing (Fig. 3). Egg volume differed among lakes, but not among perturbation levels (Table 2). *Rastrineobola argentea* from Lake Nakuwa exhibited noticeably higher fecundity, egg size, and ovary weight than all other lakes.

**Table 2:** Results from nested ANCOVAs examining variation in reproductive traits of female *Rastrineobola argentea* across lakes and perturbation levels

Trait	Effect	df	*F*	*P*	*R*^2^
Log (Fecundity)	Perturbation	2	6.20	0.003	0.83
	Lake (Perturbation)	7	11.03	< 0.001	
	Log somatic weight	1	141.09	< 0.001	
	Residual	157			
Egg volume	Perturbation	2	0.92	0.399	0.27
	Lake (Perturbation)	7	9.89	<0.001	
	Log somatic weight	1	0.11	0.739	
	Residual	151			
Log (Clutch volume)	Perturbation	2	4.79	0.010	0.73
	Lake (Perturbation)	7	10.58	< 0.001	
	Log somatic weight	1	65.69	< 0.001	
	Residual	151			
Log (Ovary weight)	Perturbation	2	11.12	< 0.001	0.82
	Lake (Perturbation)	7	14.37	< 0.001	
	Log somatic weight	1	164.02	< 0.001	
	Residual	209			

#### Across-lake trait covariation

**Figure 4:**
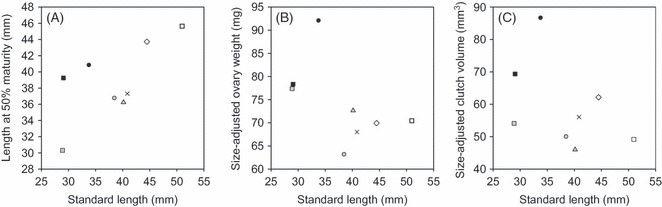
Across-lake correlations between size-adjusted trait means. The colours represent unperturbed lakes (white), lakes with Nile perch only (grey), and lakes with Nile perch and commercial fishing (black). The lakes are: Omuno (white square), Gigati (white diamond), Nawampasa (grey circle), Bisina (grey square), Meito (grey triangle), Nabugabo (grey cross), Kyoga (black circle), and Victoria (black square). Note that two lakes (Kayanja and Nakuwa) are not shown, because we did not have estimates of *L*^50^ and mean SL for these populations.

Mean standard length and *L*_50_ were positively correlated across lakes (*r* = 0.69, *P* = 0.06). *R. argentea* from unperturbed lakes tended to have both high mean body size and high *L*_50_, whereas *R. argentea* from lakes with Nile perch had both lower mean body size and lower size at maturity (Fig. 4A). There were indications of a negative relationship between body size and both metrics of total reproductive effort: size-adjusted clutch volume (*r* = −0.44, *P* = 0.28) and size-adjusted ovary weight (*r* = −0.52, *P* = 0.19). *R. argentea* from unperturbed lakes tended to have both larger mean body size and lower size-adjusted clutch volume/ovary weight, whereas populations from lakes with Nile perch and fishing tended to have lower mean body size and higher size-adjusted clutch volume/ovary weight (Fig. 4B,C). There were no significant correlations between *L*_50_ and clutch volume (*r* = 0.27, *P* = 0.57) or ovary weight (*r* = −0.0003, *P* = 0.99). Finally, there was no evidence for a trade-off in egg size versus egg number of *R. argentea* across lakes (*r* = 0.18, *P* = 0.61).

#### Assessing the effect of environmental variables

Environmental variation across lakes was summarized by three major principal component axes, which together accounted for 87% of the variation (Table C1 in Appendix S3). Inclusion of these three PCs in models of trait variation did not affect the qualitative or quantitative nature of the trends reported above. That is, perturbation remained statistically significant, and the differences between perturbation categories were conserved, regardless of whether we included lake as a nested factor (Table 2) or whether we explicitly modeled the effects of five environmental parameters that varied across those same lakes (Tables C2 and C3 in Appendix S3).

### Temporal analysis

#### Length at 50% maturity

**Figure 5:**
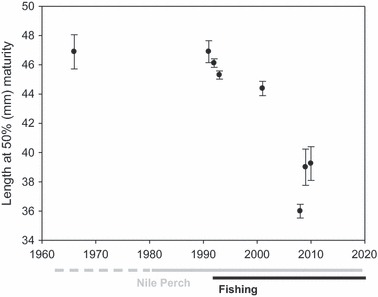
Length at 50% maturity (±1 SE) of female *Rastrineobola argentea* from the Napoleon Gulf of Lake Victoria from 1966 to 2010. The bars below the figure indicate the approximate timing of the population boom of the introduced Nile perch (grey) and the onset of commercial *R. argentea* fishing (black).

In Lake Victoria, the probability of maturation varied significantly across years (Table 1). The length at maturity of female *R. argentea* in Lake Victoria appears to have remained relatively steady at approximately 46 mm SL from 1966 until the 1990s, when it began to decline (Fig. 5). The rate of decline in *L*_50_ accelerated even further between 2000 and 2010, coinciding with intensification of the *R. argentea* fishery in the Ugandan waters of Lake Victoria. Overall, the length at maturity of female *R. argentea* has declined by about 16% in the past 44 years, which is equivalent to a phenotypic rate of change of −4.07 × 10^3^ darwins (Table 3). If we split the time series in 1991, 2 years after the opening of the *R. argentea* fishery in the Napoleon Gulf (1989), we can roughly isolate the fisheries-induced portion of the decline. Looking at this latter part of the time series, *L*_50_ has declined by about 16% in the 18 years since fishing began, which is equivalent to a rate of −10.15 × 10^3^ darwins.

**Table 3:** Mean size-adjusted trait values for *Rastrineobola argentea* from Lake Victoria at the beginning (*Z*_0_) and end (*Z*_1_) of the time-series, along with % change and rate of phenotypic change in darwins. See text for equation for estimating darwins

Trait	Time period	*Z*_0_	*Z*_1_	% Change	Rate (darwins × 10^3^)
*L*_50_ (overall)	1966 to 2008–10	46.54	39.07	−16.05	−4.07
*L*_50_ (fishing)	1991 to 2008–10	46.90	39.07	−16.70	−10.15
Relative fecundity	1966 to 2008–10	590	837	+41.91	8.14
Relative egg volume (mm^3^)	1966 to 2008–10	0.046	0.078	+68.11	12.08
Relative clutch volume (mm^3^)	1966 to 2008–10	26.05	51.42	+97.39	15.81
Relative ovary weight (mg)	1966 to 2008–10	74.44	100.48	+34.99	6.98

#### Reproductive traits

**Figure 6:**
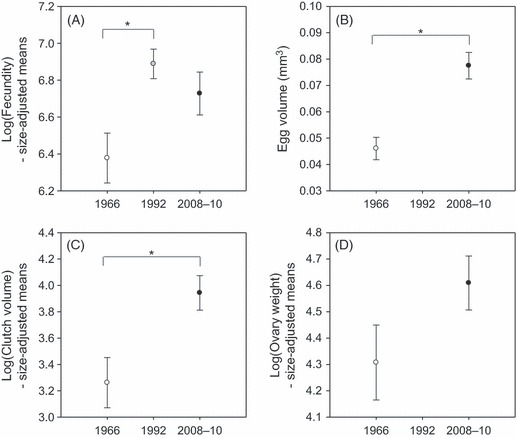
Size-adjusted means (±1 SE) for (A) fecundity, (B) egg volume, (C) clutch volume, and (D) ovary weight for mature (Stage IV–VI) female *Rastrineobola argentea* from the Napoleon Gulf of Lake Victoria. These are adjusted means from an ancova model examining variation in each trait as a function of log(somatic weight) and year. The colours indicate the following time periods: pre-Nile perch boom/pre-commercial fishing (white), post-Nile perch boom/pre-commercial fishing (grey), and post-Nile perch boom/postcommercial fishing (black). The * indicate significantly different pairs based on *post-hoc* Tukey’s tests.

In Lake Victoria, fecundity was positively correlated with body size for the 1992 and contemporary specimens (Table 4). For 1966, however, the range of body sizes of the available museum specimens was so narrow that the expected positive relationship between fecundity and standard length was not detectable. This resulted in a significant interaction between standard length and year, which we removed in order to compare adjusted means across years. Assuming a common relationship between fecundity and standard length across years, size-adjusted fecundity of *R. argentea* from Lake Victoria varied significantly across time periods (*F*_2,27_ = 6.31, *P* = 0.006), increasing from 1966 to 1992 and then declining again slightly between 1992 and 2008–2010 (Fig. 6A). Overall, size-adjusted fecundity in Lake Victoria increased about 42% from 1966 to the present, which is equivalent to 8.14 × 10^3^ darwins (Table 3). In Lake Kyoga, fecundity was also positively correlated with standard length, and there were no significant differences in the slope of this relationship between years (Table 4). Size-adjusted fecundity did not differ significantly before (1991) vs after (2008–2010) the development of a commercial *R. argentea* fishery in Lake Kyoga (*F*_1,76_ = 0.006, *P* = 0.939).

**Table 4:** Results from ANCOVAs examining variation in fecundity, egg volume, clutch volume, and ovary weight of female *Rastrineobola argentea* in the Napoleon Gulf of Lake Victoria from 1966 to 2008–2010; as well as variation in fecundity of *R. argentea* in Lake Kyoga from 1991 to 2008–2010. SL refers to standard length (mm)

Lake	Trait	Effect	df	*F*	*P*	*R*^2^
Victoria	Log(Fecundity)	Year	2,27	3.19	0.057	0.44
		SL	1,27	0.99	0.330	
		Year × SL	2,27	3.91	0.032	
Victoria	Log(Egg volume)	Year	1,33	15.80	< 0.001	0.29
		Somatic weight	1,33	5.83	0.022	
Victoria	Log(Clutch volume)	Year	1,33	6.70	0.014	0.19
		Somatic weight	1,33	10.09	0.003	
Victoria	Log(Ovary weight)	Year	1,34	2.27	0.141	0.44
		Somatic weight	1,34	23.34	<0.001	
Kyoga	Log(Fecundity)	Year	1,76	0.006	0.939	0.86
		Log(SL)	1,76	154.25	<0.001	

All egg traits (egg volume, clutch volume, and ovary weight) were significantly positively correlated with somatic body weight, and this relationship was consistent across time periods (Table 4). Size-adjusted egg volume, clutch volume, and ovary weight of *R. argentea* in the Napoleon Gulf of Lake Victoria all showed indications of an increase between 1966 and 2008–2010 (Fig. 6B–D), and this increase was statistically significant for the first two traits (Table 4). Rates of increase for these traits varied between 6.98 × 10^3^ and 15.81 × 10^3^ darwins (Table 3).

## Discussion

We examined the joint effects of an introduced predator and fishing on life history traits in *R. argentea*, using both spatial (across-lake) and temporal comparisons. We found, first, that the mean length of *R. argentea* was significantly lower in invaded and fished lakes relative to unperturbed lakes and that these differences were conserved across multiple sampling years. Length at 50% maturity was also significantly reduced in perturbed relative to unperturbed contexts. In the spatial analysis, *L*_50_ was lowest in lakes with Nile perch only, whereas in the temporal analysis, *L*_50_ was lowest in years with Nile perch and fishing. Size-specific fecundity generally increased in perturbed relative to unperturbed contexts. In the spatial analysis, the greatest increase in fecundity was observed in lakes with Nile perch and fishing, whereas in the temporal analysis, the greatest increase in fecundity was observed after the Nile perch introduction (1992) and there was no additional increase in fecundity after almost two decades of commercial fishing, in either Lake Victoria or Lake Kyoga. Egg volume did not differ across perturbation levels spatially, but did increase significantly over time. Size-adjusted clutch volume and ovary weight both increased in perturbed relative to unperturbed contexts, being greatest in contexts with introduced Nile perch and fishing, both across lakes and through time. Overall, introduced Nile perch and fishing tended to be associated with a suite of life history responses in *R. argentea*, including decreased body size, maturation at smaller sizes, and increased reproductive effort (larger eggs; and higher relative fecundity, clutch volume, and ovary weight).

Although it is not possible to conclusively infer causation from correlative, field-based studies, we believe that this study provides strong support for the hypothesis that the Nile perch introduction and commercial fishing are important drivers of contemporary life history change in *R. argentea*. First, we found that patterns of covariation between life history phenotypes and the presence/absence of Nile perch and fishing were quite consistently replicated across both space and time. Such congruence would be unlikely if other unmeasured variables were the major drivers of life history change. Second, in the spatial analysis, we showed that our main findings (i.e., significant differences among perturbation categories) were robust to the inclusion of five important environmental variables (lake area, lake depth, water temperature, dissolved oxygen, and water transparency). It is important to note here that lake area is somewhat confounded with the effect of fishing, given that *R. argentea* fisheries have only been developed on the two largest lakes (Victoria and Kyoga). However, lake area tends to be positively correlated with body size and size at maturity in fish (e.g., Burr 1993; Chen and Harvey 1994; McDermid et al. 2010). If lake size were a major driver in our system, we would expect *R. argentea* in lakes Kyoga and Victoria to mature at larger sizes and be larger overall relative to *R. argentea* in smaller lakes; however, we observed the opposite. Third, the observed direction of change in life history traits is consistent with predictions from life history theory, that is, that increased mortality will select for earlier maturation at smaller sizes (Law and Grey 1989; Abrams and Rowe 1996; Ernande et al. 2004).

### *Rastrineobola argentea* as a survivor in the highly perturbed Lake Victoria ecosystem

This study greatly expands our understanding of how *R. argentea* has adapted to novel stressors in Lake Victoria. Wanink (1998) was the first to suggest that *R. argentea* may have undergone rapid phenotypic changes as a result of the Nile perch introduction. He documented striking changes in life history traits of *R. argentea* in the Mwanza Gulf of Lake Victoria before (1983) versus after (1987–88) the Nile perch population boom, including declines in the mean size of ripe females, age and size at maturity, and absolute fecundity (Wanink 1998; Wanink and Witte 2000). By providing a longer-term historical context and covering a broader spatial scale, our data help to further elucidate the generality, timing, and potential drivers of these trends from the Tanzanian waters. For example, Wanink (1998) interpreted the decline in size at maturity of female *R. argentea* in the Mwanza Gulf between 1983 and 1988 primarily as a consequence of increased Nile perch predation. However, historical catch landings data show that this time period also coincided with the development of the *R. argentea* fishery in the Tanzanian waters (Wanink 1999). Interestingly, the difference in the timing of the decline in *L*_50_ of *R. argentea* across Lake Victoria is consistent with the difference in the timing of the development of the *R. argentea* fishery (both occurred about a decade earlier in the Mwanza Gulf relative to the Napoleon Gulf). Taken together, Wanink and Witte’s data and our own show that a significant decline in *L*_50_ in *R. argentea* has occurred in at least two regions of Lake Victoria and suggest that commercial fishing has likely played an equal, if not more important, role than Nile perch predation in driving these changes.

Because the *R. argentea* fishery is still so new, there has been little research prior to this study specifically examining its potential phenotypic impacts. Wandera (1992) found a small decline in the mean size of adult *R. argentea* during early years of expansion of the fishery in Ugandan waters. Further declines in the mean length of the Ugandan population were noted between the 1990s and 2006 (NaFIRRI, 2008). Taabu (2004) found that *R. argentea* in heavily fished inshore areas of Northern Lake Victoria were significantly smaller than *R. argentea* from offshore areas, consistent with our findings that populations are significantly smaller in fished versus unfished lakes. Previously published values for *L*_50_ of *R.* argentea from the Northern waters of Lake Victoria hint at a decline as well, from 43 to 44 mm SL in 1988 (Wandera 1992), to 42 mm SL in 1996–97 (Wandera 1999), 41 mm in 2001–2002 (Taabu 2004), to 40 mm SL in 2004–2005 (NaFIRRI, 2005). Our study is consistent with, and extends, these early trends.

### Underlying mechanisms for life history changes in *Rastrineobola argentea*

What mechanisms might be underlying these observed shifts in life history traits in *R. argentea*? One possibility is that they represent a genetic (evolutionary) response to selection imposed by fishing and/or Nile perch predation. Genetic changes can be expected if mortality is high and nonrandom and the traits under selection are heritable. Artificial selection experiments and aquaculture studies have shown that life history traits are moderately heritable in many fishes (Gjedrem 1983; Law 2000), although this has not been tested in *R. argentea* specifically. We do know, however, that fishing mortality on *R.* *argentea* is high (1.22–1.98/year, Manyala and Ojuok 2007) and that this species has a short generation time (estimates from the Tanzanian waters of Lake Victoria: 0.3–0.9 year, Wanink 1998), making contemporary evolutionary change a plausible hypothesis. A second possibility is that the observed shifts in life history traits represent plastic changes occurring as an indirect result of fishing and/or Nile perch predation. In theory, fishing and/or predation can lower the density of prey populations, thus increasing per capita food availability. This can result in increased individual growth rates, and associated plastic changes in life history traits, such as earlier maturation, larger size at maturity, and increased fecundity (e.g., Cardinale and Modin 1999; Cassoff et al. 2007; Walsh and Reznick 2008). In contrast to this expectation, however, there is evidence that the biomass of *R. argentea* in Lake Victoria has increased dramatically over the past three decades, potentially due to competitive release following the decline of the haplochromine cichlids in the 1980s (Wanink and Witte 2000). Experimental catch rates of *R. argentea* in the Mwanza Gulf increased sevenfold from 1981 to 1989 (Wanink 1999) and estimated lake-wide biomass more than quadrupled from 245 000 tons in 1999 to 1 055 600 tons in 2007 (Tumwebaze et al. 2007; NaFIRRI, 2008). There are few data on how availability of zooplankton (a primary food of *R. argentea*) has changed over time in Lake Victoria, but it may have increased as well, given that algal biomass has quintupled since the 1960s (Hecky et al. 2010) and that most of the lake’s other indigenous zooplanktivores have gone extinct or dramatically declined (Witte et al. 1992), although there is evidence of a limited, contemporary resurgence of some zooplanktivorous cichlids (Witte et al. 2000). Thus, individual growth rates of *R. argentea* may conceivably have increased (if food availability has increased more rapidly than biomass), decreased (if the converse is true), or remained the same. Unfortunately, owing to the difficulty of aging tropical fish, there are no direct estimates of how individual growth rates of *R. argentea* vary through time or across lakes in the Lake Victoria basin. Length-frequency analysis has been used to derive growth estimates in two earlier studies (Wandera and Wanink 1995; Wanink 1998); however, interpretation of these data is challenging, and there is clearly a need for additional aging studies.

Our findings for *L*_50_ (lower *L*_50_ through time, and in invaded relative to uninvaded lakes) are consistent then with two possible mechanisms: (i) an evolutionary response to selection for smaller size at maturity, and/or (ii) a plastic effect, for example, resulting from reduced per capita food availability. The observed pattern for reproductive effort (higher through time, and in fished versus unfished contexts) would be also consistent with two possibilities: (i) an evolutionary response to selection for increased reproductive investment, and/or (ii) a plastic effect, for instance, resulting from increased per capita food availability. Overall, the life history changes that we observed likely reflect some combination of evolutionary change and phenotypic plasticity, but our data do not yet allow us to determine the relative importance of these two mechanisms. Further research, such as common garden experiments rearing fish from invaded and uninvaded lakes at multiple resource levels, is necessary to distinguish between these various possibilities.

We should note here that interpretations regarding the observed changes in *L*_50_ will depend on the form (shape) of the probabilistic maturation reaction norm (PMRN) for *R. argentea.* PMRNs describe the age and size-specific probabilities of reaching sexual maturation, independent of growth and mortality (Heino et al. 2002). Our interpretations above are based on the assumption that the PMRN for *R. argentea* has a negative slope, that is, that fish will have the same probability of maturing either by being large (at a young age) or by being old (at a smaller size). Of the species for which PMRNs have been calculated so far, almost all do indeed have negative slopes (e.g., North Sea plaice (Grift et al. 2003; van Walraven et al. 2010); Northern cod (Olsen et al. 2004); American plaice (Barot et al. 2005); North Sea sole (Mollet et al. 2007); and Icelandic cod (Pardoe et al. 2009)), making this a reasonable starting assumption for *R. argentea*. It should be pointed out too, however, that fishing can cause the slope of the PMRN itself to evolve, which has been highlighted in theoretical models (Ernande et al. 2004) and demonstrated empirically (Engelhard and Heino 2004). Elucidating the form of the PMRN for *R. argentea* and examining temporal and spatial variation in PMRNs will be an important direction for future research on fisheries-induced life history change in this species.

### Fisheries-induced life history change

This study represents one of the first well-documented examples of fisheries-induced phenotypic change in a tropical, freshwater species. Our results corroborate trends observed in many other commercially harvested fish stocks, where declines in mean length and length at maturity have been widespread (reviewed in Hutchings and Baum 2005; Jørgensen et al. 2007; Sharpe and Hendry 2009). For *R. argentea*, we found a rate of fisheries-associated decline in *L*_50_ of −10.15 × 10^3^ darwins, which is very close to the average rate of decline for other commercial stocks (mean of 18 stocks: −10.6 + −9.6 × 10^3^ darwins (Sharpe and Hendry 2009)). We should note, however, that the estimated generation time for *R. argentea* (0.3–0.9 year, Wanink 1998) is much shorter than all other stocks in that meta-analysis, which should be kept in mind when comparing rates across taxa. Fewer studies have examined the long-term effects of fishing on fecundity or reproductive investment. Increases in size-specific fecundity following several decades of exploitation have been reported for inshore North Sea cod, *Gadus morhua* (Yoneda and Wright 2004), North Sea haddock, *Melanogrammus aeglefinus* (Wright et al. 2011), and Lake Constance whitefish, *Coregonus lavaretus* (Thomas et al. 2009), but were not conclusive for North Sea plaice, *Pleuronectes platessa* (Rijnsdorp et al. 2005; van Walraven et al. 2010).

The fact that the rate of fisheries-induced change in *R. argentea* is comparable to many heavily fished marine stocks is remarkable, for several reasons. First, the duration of exploitation for *R. argentea* has been quite short (about 20 years in the Ugandan waters), relative to many North Atlantic stocks that have been exploited for hundreds of years. Second, the *R. argentea* fishery in Ugandan waters is operated primarily from paddled craft, in contrast to many highly industrialized marine fisheries. Third, the *R. argentea* fishery targets a very broad range of sizes that includes both mature and immature individuals (Wandera 1992; Taabu 2004; D. M. T. Sharpe and L. J. Chapman, unpublished data) in contrast to many commercial marine fisheries that operate using highly size-selective gears. This last point is of particular interest, because age or size-selective mortality has long been thought to be an important prerequisite for life history evolution in general (Gadgil and Bossert 1970; Law 1979; Michod 1979) and for fisheries-induced evolution in particular. However, recent models suggest that unselective predation (Abrams and Rowe 1996) or fishing (Law and Grey 1989; Heino 1998; Ernande et al. 2004) can also cause evolution in life history traits. Our work adds further empirical support to this body of theory and suggests that strong size-selectivity may not be a necessary prerequisite for phenotypic change in life history traits in commercially fished stocks, so long as mortality is high.

### Predator-induced life history change

This study is also one of the first to document contemporary life history change in native prey in response to an introduced predator. In a recent review of evolutionary responses of natives to introduced species, Strauss et al. (2006) compiled 11 known cases of phenotypic change in native prey in response to introduced predators: of these, six involved behavioral changes in the prey, and five involved changes in morphology, but there were none reporting changes in life history traits. To our knowledge, only a few other studies to date have found evidence for life history change in native prey following exposure to an introduced predator (e.g., Fisk et al. 2007; Billman et al. 2011). As more examples like these accumulate, we will be better able to understand the conditions under which native prey adapt to novel predators rather than declining to extinction.

### Implications for sustainability of the *Rastrineobola argentea* fishery

Previous authors have argued that *R. argentea’s* life history tactics and flexibility in other traits facilitated its success relative to other prey of the Nile perch, many of which went extinct (Wanink 1998; Wanink and Witte 2000). Indeed, *R. argentea* is a classic example of r-selected (*sensu*Pianka 1970) or opportunistic (*sensu*Winemiller and Rose 1992) species, which are expected to be resilient to uncertain environments and high mortality. This logic underlies the widespread assumption that short-lived, fast-growing pelagic fishes should be more resistant to overfishing than long-lived, late-maturing species. However, a recent meta-analysis showed that globally, fisheries targeting small, low trophic level species are just as likely to collapse as those targeting large, high trophic level fishes (Pinsky et al. 2011). Should we be concerned then about the long-term sustainability of the *R. argentea* fishery?

Based on our findings that (i) mean body size is 34% lower in invaded and fished lakes relative to unperturbed lakes; and (ii) *L*_50_ has declined by 16% since the Nile perch introduction and onset of fishing, we believe that there is now reason for concern for the future of the *R. argentea* fishery. Several researchers have suggested that rapid life history changes should be interpreted as a warning sign of impending stock decline, as they typically signal high levels of mortality (Trippel 1995; Olsen et al. 2004). Indeed, fishing mortality for *R. argentea* has been estimated to be as high as 1.22–1.98/year (Manyala and Ojuok 2007). Furthermore, both theory and experiment suggest that fisheries-induced changes in life history traits can have negative implications for yield and the probability of population persistence (Edley and Law 1988; Heino 1998; Conover and Munch 2002; Walsh et al. 2006). In the case of *R. argentea*, the dramatic decline in body size and *L*_50_ over time and in perturbed relative to unperturbed lakes is of greatest concern, because fecundity (and hence recruitment) is positively correlated with the body size of spawners. Even though size-specific fecundity has increased, it may not be enough to counteract the detrimental effects of the overall decline in body size, resulting in a net decline in per capita recruitment.

*Rastrineobola argentea* is now the most important commercial fish stock by mass in Lake Victoria (NaFIRRI 2008), whose combined fisheries meet the fish consumption needs of an estimated 30 million East Africans (LVFO 2011). The Lake Victoria basin is also home to the fastest growing human population on the African continent (UNEP 2006), so ensuring food security in the coming decades will be a challenge. The *R. argentea* fishery is still in its infancy, yet already shows signs of potentially detrimental fisheries-induced phenotypic changes. Given the long history of overfishing and previous fisheries collapses in Lake Victoria, we urge that these early indications of fisheries-induced change in *R. argentea* be investigated further. We recommend continued monitoring of life history traits in *R. argentea*, as well as the implementation of a permanent lake-wide initiative to monitor basic stock characteristics. To reduce the probability of further fisheries-induced life history change in *R. argentea*, there are two avenues for managers to consider: (i) altering catch selectivity so as to reduce selection for early maturing genotypes, and/or (ii) lowering the overall rate of fishing mortality.

In the first instance, many authors have argued that the dome-shaped selectivity curves that characterize many fixed gears such as traps and gill-nets (those that target intermediate-sized fish and provide protection for both the smallest and largest individuals) are preferable in terms of avoiding fisheries-induced evolution (Conover and Munch 2002; Law 2007; Jørgensen et al. 2009; Hutchings 2009). In contrast, the knife-edged selectivity curves that characterize many active gears like trawls are most likely to generate strong selection for earlier maturation (Jørgensen et al. 2009; Hutchings 2009). Given that the *R. argentea* fishery currently relies on trawling with small-mesh gears exclusively, what options may be available to minimize the strong selection that this gear likely generates? Models indicate that fisheries-induced evolution imposed by knife-edged selectivity curves can be mitigated only by keeping *F* very low and confined to mature fish (Jørgensen et al. 2009), that is, by setting the minimum size threshold above the maturation reaction norm (Ernande et al. 2004). In the case of *R. argentea*, this could be achieved by switching back to a 10-mm mesh lampara net, which primarily targets individuals above 40 mm SL (just above the current size at maturation of *R. argentea* in Lake Victoria), and/or by encouraging fishers to move further offshore where there are fewer immature fish (Wanink 1999; Taabu 2004). In addition to reducing the probability of further fisheries-induced change, such practices would be consistent with the traditional fisheries management goal of preventing recruitment overfishing by reducing the high proportion of immature *R. argentea* in commercial catches (Wanink 1999; Taabu 2004; Tumwebaze et al. 2007). They would also help reduce bycatch of juveniles of other commercially important species such as Nile perch and Nile tilapia (*Oreochromis niloticus*) (Taabu 2004).

A second avenue for avoiding fisheries-induced change irrespective of modifications to gear selectivity would be simply to lower the overall rate of fishing mortality (Hutchings 2009). Further research aimed at identifying the evolutionarily sensitive threshold for this stock (i.e., the level of fishing mortality above which fisheries-induced evolution is expected to occur, F_evol_, (Hutchings 2009)) would be useful for determining whether fishing effort should be curbed. In the Lake Victoria basin, this could be achieved by limiting the number of boats and/or lampara nets at each landing, although this would be challenging to implement in practice.

## Data archiving statement

Data deposited in the Dryad repository: doi:10.5061/dryad.1154dd89.

## Supporting information

**Appendix S1.** Study sites.

**Appendix S2.** Data sources for historical analysis.

**Appendix S3.** Assessing the effect of environmental variables.
