# Supplementary material for: Life history change in response to fishing and an introduced predator in the East African cyprinid Rastrineobola argentea
Source: Evol Appl. 2012 Nov;5(7):677–93. doi: 10.1111/j.1752-4571.2012.00245.x (PMC3492894; doi:10.1111/j.1752-4571.2012.00245.x)
Supplement: Supplementary file 1 [file eva0005-0677-SD1.pdf]

Appendix A: Study Sites

Table A1

Lake	Years Sampled	Location	Surface area (km^2)	Mean Depth (m)	Elevation (m)	Secchi Depth (m)	Temperature (°C)	Dissolved Oxygen (mg/L)	Nile Perch Introduced	*R. argentea* Fishery	Native piscivores (predation on *R. argentea?*)
Victoria	2008-10	N00°24'52.7"<br>E 033°12'26.9"	63.50<br>(68,800)	10.40<br>(40)	1136	1.17<br>± 0.05	27.20<br>± 0.50	7.68<br>± 0.50	Yes<br>(1954, 1960s)	Yes<br>(1989)	S.v (no)[1]
Nabugabo	2008-10	S00°21'24.38"<br>E31°52'26.02"	33.99	4.53	1151	0.73<br>± 0.04	24.62<br>± 0.21	6.51<br>± 0.36	Yes<br>(1960,1963)	Minimal<br>(2011)	S.i. (yes)[1,2]
Kayanja	2009-10	S00°16'30.85"<br>E31°52'10.16"	1.25	2.61	1165	0.50<br>± 0.02	25.33<br>± 0.08	7.68<br>± 0.33	No	No	C.g. (no)[1,2], G.v (yes)[1,2]
Kyoga	2008-10	N01°18'40.3"<br>E 033°18'22.4"	1971.35<br>(2047)	2.46	1033	0.61<br>± 0.06	30.56<br>± 0.56	10.48<br>± 0.27	Yes<br>(1955)	Yes<br>(1995)	C.g (yes)[1], S.v. (yes)[1,3], S.i. (no)[1,3]
Nawampasa	2009-10	N01°16'59.0"<br>E 033°21'35.6"	8.53	1.62	1029	0.48<br>± 0.02	29.65<br>± 0.26	6.61<br>± 0.24	Yes<br>(1998)	No	C.g. (no)[1,4], S.v. (no)[1,3]
Nakuwa	2009-10	N01°13'07.8"<br>E 033°27'18.5"	95.79	2.22	1034	0.38<br>± 0.02	31.27<br>± 0.44	4.85<br>± 0.72	Yes<br>(1970s)	No	C.g. (no)[1,4], S.i.(no)[1,3,4], S.v. (no)[1,3]
Gigati	2009-10	N01°16'12.3"<br>E 033°33'44.4"	7.52	2.03	1035	0.62<br>± 0.03	28.83<br>± 0.61	7.02<br>± 0.48	No	No	None[4]
Meito	2010	N01°17'09.4"<br>E 033°33'56.7"	14.64	2.79	1034	1.35<br>± 0.08	26.90<br>± 0.31	5.52<br>± 0.43	Yes	Minimal<br>(2005)	S.v.(unknown)[4]
Omuno	2010	N01°16'00.3"<br>E 033°38'45.8"	5.56	1.37	1025	0.26<br>± 0.00	28.87<br>± 0.23	8.09<br>± 0.12	No	No	C.g.(unknown)[4]
Bisina	2009-10	N01°37'26.1"<br>E 033°55'21.4"	349.31	3.86	1043	1.96<br>± 0.08	27.81<br>± 0.17	6.63<br>± 0.47	Yes<br>(1970s)	No	C.g. (no)[1,4]

Table A1. Physical attributes of the 10 lakes sampled for this study. Geographic co-ordinates and elevation refer specifically to the landing site on each lake from which our sampling was based. Surface area was estimated from satellite images downloaded from Google Earth. Mean depth was estimated from three replicate depth measurements taken during our 2010 field survey at sites sampled. For Lake Victoria, we specify the surface area and depth for the Napoleon Gulf, where all of our sampling was carried out, and for Lake Victoria as a whole (in parentheses). For Lake Kyoga, we speficy the surface area for the main lake where we carried out our sampling, as well as the entire surface area including additional branches (in parentheses). The values for Secchi depth, water temperature and dissolved oxygen are the means (± 1 SE) of three replicates taken at the surface in the afternoon at each sampling site. For the Nile perch, the approximate year of introduction is indicated in parentheses. For *the R. argentea* fishery, the approximate year that the fishery started is indicated in parentheses. The *R. argentea* fisheries on lakes Nabugabo and Meito are considered minimal because only 1 or 2 fishers are active, and only sporadically. The final column indicates the presence/absence of 4 native piscivores known to sometimes feed on *R. argentea*: *Clarias gariepinus* (C.g), *Gnathonemus victoriae* (G.v.), *Schilbe intermedius* (S.i.), and *Synodontus victoriae* (S.v.). Where data are available, we have indicated whether these fish species are known to feed on *R. argentea* in the lake in

question (yes, no, or unknown). References for the distribution and diet of the native piscivores: (1) Mbabazi (2004); (2) Namulemo, et al. unpl. data; (3) Schwartz et al. (2006); (4) Sharpe and Chapman, unpl. data

Appendix B: Data sources for historical analysis

Table B1

Lake	Year	Site	n (females)	Traits	Source
Victoria	1966	Napoleon Gulf	39	L_{50}, fecundity, egg traits	NaFIRRI Museum
Victoria	1989	Napoleon Gulf	204	L_{50}	S.B. Wandera, unpl. data
Victoria	1991	Napoleon Gulf	174	L_{50}	S.B. Wandera, unpl. data
Victoria	1992	Napoleon Gulf	1075	L_{50}, fecundity	S.B. Wandera, unpl. data
Victoria	1993	Napoleon Gulf	1012	L_{50}	S.B. Wandera, unpl. data
Victoria	2000/2003	Napoleon Gulf	35	L_{50}	NaFIRRI Museum
Kyoga	1991	Bukungu Landing	44	fecundity	S.B. Wandera, unpl. data

Table B1 Historical data and specimens of *R. argentea* available from lakes Victoria and Kyoga.

Appendix C: Assessing the effect of environmental variables

Table C1

	PC1 (35.3%)	PC2 (32.1%)	PC3 (19.3%)
Lake area	-0.02	**0.74**	-0.20
Lake depth	**0.58**	0.24	0.24
Secchi depth	**0.59**	0.26	-0.38
Temperature	**-0.54**	0.36	-0.41
Dissolved oxygen	-0.18	0.44	**0.77**

Table C1. Results of a principal components analysis on the 5 environmental variables measured for each lake (lake area, lake depth, Secchi depth, temperature and dissolved oxygen). The variance explained by each principal component is indicated in parentheses. The values shown are the loadings of each environmental variable onto each of the first three principal component axes. The variables that loaded most heavily onto each component (>0.50) are indicated in bold.

Table C2

	Effect	df	F	p	R^2
Standard Length	Perturbation	2	216.61	< 0.001	0.60
	Env PC1	1	327.93	< 0.001	
	Env PC2	1	141.01	< 0.001	
	Env PC3	1	190.33	< 0.001	
	Residual	2477			
Log Fecundity	Perturbation	2	5.95	0.003	0.77
	Log Somatic Weight	1	381.13	< 0.001	
	Env PC1	1	5.11	0.025	
	Env PC2	1	3.96	0.048	
	Env PC3	1	1.40	0.239	
	Residual	161			
Egg Volume	Perturbation	2	6.78	0.001	0.23
	Log Somatic Weight	1	1.01	0.317	
	Env PC1	1	0.30	0.584	
	Env PC2	1	0.30	0.582	
	Env PC3	1	37.55	<0.001	
	Residual	155			
Log Clutch Volume	Perturbation	2	5.27	0.006	0.73
	Log Somatic Weight	1	194.77	< 0.001	
	Env PC1	1	4.74	0.031	
	Env PC2	1	3.33	0.070	
	Env PC3	1	14.80	< 0.001	
	Residual	155			
Log Ovary Weight	Perturbation	2	10.10	< 0.001	0.82
	Log Somatic Weight	1	490.56	< 0.001	
	Env PC1	1	5.53	0.020	
	Env PC2	1	8.22	0.005	
	Env PC3	1	28.08	< 0.001	
	Residual	213			

Table C2. Results of a series of general linear models examining the effects of perturbation level and environmental variables (first 3 PCs, Table C2) on life history traits.

Table C3

	Effect	df	Deviance	p
L_{50}	Perturbation	2	208.32	< 0.001
	Standard Length	1	430.68	< 0.001
	Env PC1	1	48.60	< 0.001
	Env PC2	1	61.56	< 0.001
	Env PC3	1	34.84	< 0.001

Table C3. Results of a general linear model that examined the effects of perturbation level and environmental variables (first 3 PCS, Table C2) on L_{50}.

Literature Cited in Appendix:

Mbabazi, D. 2004. Trophic characterization of the dominant fishes in the Victoria and Kyoga lake basins.

Schwartz, J. D. ., Pallin, M. J., Michener, R. H., Mbabazi, D., & Kaufman, L. 2006. Effects of Nile perch, Lates niloticus, on functional and specific fish diversity in Uganda's Lake Kyoga system. *African Journal of Ecology* **44**: 145-156.
